# Angiotensin receptor blockers, but not angiotensin-converting enzyme inhibitors, inhibit abnormal bone changes in spondyloarthritis

**DOI:** 10.1038/s12276-023-01103-z

**Published:** 2023-11-01

**Authors:** Jin Sun Choi, Ji-Young Kim, Min-Joo Ahn, Hanbit Jang, Seungtaek Song, Sung Hoon Choi, Ye-Soo Park, Sungsin Jo, Tae-Hwan Kim, Seung Cheol Shim

**Affiliations:** 1 Division of Rheumatology, Regional Rheumatoid & Degenerative Arthritis Center, Chungnam National University Hospital, Daejeon, 35015 Republic of Korea; 2 Department of Orthopaedic Surgery, Hanyang University Hospital, Seoul, 04763 Republic of Korea; 3 Department of Orthopedic Surgery, Guri Hospital, Hanyang University College of Medicine, Guri, 11923 Republic of Korea; 4 Hanyang University Institute for Rheumatology Research (HYIRR), Seoul, 04763 Republic of Korea; 5 Department of Rheumatology, Hanyang University Hospital for Rheumatic Diseases, Seoul, 04763 Republic of Korea

**Keywords:** Ankylosing spondylitis, Extracellular signalling molecules

## Abstract

Spondyloarthritis (SpA) is a chronic inflammatory disease that results in bone ankylosis. The tissue renin-angiotensin system (RAS) is an emerging pathway potentially implicated in SpA-associated bone changes. The aim of the present study was to determine the mechanisms underlying this relationship. Sakaguchi (SKG) mice injected with curdlan (SKGc), animal models for SpA, were treated with RAS modulators, angiotensin II receptor blockers (ARBs) or angiotensin-converting enzyme inhibitors (ACEis). Disease activity was assessed using clinical scores and computed tomography scans. Mouse primary bone marrow monocytes (BMMs), osteoblast (OB) progenitor cells, peripheral blood monocytes (PBMCs), and bone-derived cells (BdCs) from patients with radiographic axial SpA (r-axSpA) were used to investigate the role of RAS in SpA pathogenesis. The expression of RAS components was significantly increased in SKGc mouse joints, and ARBs significantly reduced erosion and systemic bone loss, whereas ACEis did not. Osteoclast (OC) differentiation from primary BMMs, mediated by TRAF6, was inhibited by ARBs but promoted by ACEis; the modulators also exerted opposite effects on OB differentiation. Expression of RAS molecules was higher in PBMCs and BdCs of patients with r-axSpA than in control participants. ARBs inhibited OB differentiation in the BdCs of patients with r-axSpA, whereas ACEis did not. Neither ARBs nor ACEis affected OB differentiation in the control participants. In SpA, a condition characterized by RAS overexpression, ARBs, but not ACEis, inhibited OC and OB differentiation and bone progression. The findings should be taken into account when treating patients with SpA using RAS modulators.

## Unraveling Spondyloarthritis: A Study on RAS Overexpression

Researchers have discovered that the renin-angiotensin system (RAS) plays a key role in bone changes associated with spondyloarthritis, a chronic inflammatory disease. The study found that RAS components were overexpressed in patients with the disease, and that angiotensin receptor blockers (ARBs) significantly reduced bone erosion and increased bone mineral density in an animal model of spondyloarthritis. In contrast, angiotensin-converting enzyme inhibitors (ACEis) increased osteoblast and osteoclast differentiation, leading to higher bone formation and erosion. The findings suggest that RAS modulation may be an effective therapeutic target for spondyloarthritis, with ARBs potentially being more beneficial than ACEis for treating hypertension in patients with the condition.

## Introduction

Spondyloarthritis (SpA), a chronic inflammatory disease, is characterized by a unique bone phenotype resulting from a simultaneous increase in bone formation and resorption^1,2^, culminating in skeletal damage and functional impairment^3,4^. Although long-term use of tumor necrosis factor blockers^5,6^ may reduce radiographic progression, particularly in the early phase of the disease, novel therapies targeting bone changes in SpA are needed.

The renin-angiotensin system (RAS) is classically viewed as an endocrine system that regulates hemodynamic equilibrium, circulating volume, and electrolyte balance^7^. Angiotensinogen, which is secreted by the liver, is cleaved by renin, an enzyme produced in the kidneys, to form angiotensin (Ang I). Ang I is then cleaved by angiotensin-converting enzyme (ACE) to form Ang II, an effector hormone that acts through its classic receptor, Ang II receptor 1 (AT1R)^8^. RAS modulators, such as ACE inhibitors (ACEis) and angiotensin receptor blockers (ARBs), are widely used to treat hypertension and heart and kidney diseases^9–14^.

Components of the alternative RAS axes, such as ACE2 and Ang 1-7, which challenge the standard view of RAS, have recently been identified^15^. ACE2, an enzyme that converts Ang II to Ang 1-7^16^, has been studied extensively in recent years due to its role as an entry point for coronavirus disease^17,18^. Ang 1-7 is also generated by neprilysin (neutral endopeptidase, NEP) from Ang I^19^.

Along with the classic pathway, components of the RAS are expressed and exert specific local effects in multiple tissues, a phenomenon referred to as tissue RAS^7,20^. RAS components, particularly the major effector protein Ang II and its receptors, are expressed in the local milieu of bones and regulate bone metabolism^21^. Animal studies have demonstrated that tissue RAS plays a key role in postmenopausal^22^, age-related^23^, and glucocorticoid-induced osteoporosis^24^. In addition, RAS components expressed by T cells, natural killer cells, macrophages, and dendritic cells are involved in immune activation^25^. Tissue RAS is also involved in chronic inflammatory disorders, such as systemic lupus erythematosus^26^ and rheumatoid arthritis (RA)^27^. A recent study on an animal model of RA showed that Ang II exacerbates bone erosion^28^. However, the effect of RAS modulators on bone cells and the role of RAS in SpA are yet to be explored.

Therefore, in the present study, an in vivo model of SpA, an in vitro culture system of osteoclasts (OCs) and osteoblasts (OBs), and human samples were used to investigate the potential role of RAS in abnormal bone changes induced by SpA, the different effects of each RAS modulator, and the mechanisms by which RAS modulators affect bone cell differentiation.

## Materials and methods

### Experimental mouse model and clinical scoring

Female SKG mice were purchased from CLEA Japan, Inc. (Tokyo, Japan), and were maintained under specific pathogen-free conditions. Eleven-week-old female SKG mice were intraperitoneally injected with 3 mg curdlan (Wako, Osaka, Japan; SKGc). The experimental animals were divided into four groups: negative control (*n* = 5 mice), vehicle (saline, *n* = 15), losartan treatment (*n* = 12; SK Chemicals, South Korea), and enalapril treatment (*n* = 10; Chong Kun Dang Pharmaceutical Corp., South Korea). Losartan, an AT1R blocker, and enalapril, an ACEi (10 mg/kg each), were administered to the treatment groups for five days a week, from Day 0 to 56^29,30^. The clinical signs of the mice were monitored twice per week and scored by two independent observers. The severity of arthritis was assessed via the clinical arthritis score and myeloperoxidase (MPO) activity obtained using an in vivo imaging system (IVIS) with a XenoLight RediJect inflammation probe (PerkinElmer, Inc., Waltham, MA, USA). Clinical arthritis scores were evaluated on a scale of 0 to 4 for each limb (0, no swelling; 1, slight swelling and erythema; 2, moderate swelling and erythema; 3, severe swelling and erythema; and 4, maximal inflammation with joint rigidity). Thus, the maximum possible score for each mouse was 16^31^.

### Analysis of bony changes in the ankle joints of SKGc mice

Bone deformities in the ankle joints of the mice were analyzed using Quantum FX micro-computed tomography (CT) (PerkinElmer, Inc.). The micro-CT settings used were as follows: energy/intensity of 90 kVp, electric current of 160 µA, sampling time of 3 min, and field of view of 5 mm. Three-dimensional views were constructed using 3D Viewer imaging software (PerkinElmer, Inc.).

Erosions were defined as clear juxta-articular breaks in the cortical shell. Osteophytes were defined as bony protrusions of the juxta-articular cortical shell. Erosions and osteophytes were evaluated on the following scale: 0, no surface changes; 1, surface changes on <25% of the surface; 2, surface changes on >25% and <50% of the surface; and 3, surface changes on >50% of the surface^32^.

### Patient samples and human primary bone-derived cells (BdCs)

All studies involving human materials were performed in compliance with the Helsinki Declaration and approved by the Ethics Committee of Chungnam University Hospital and Hanyang University Hospital; written informed consent was obtained from all subjects (IRB-2015-03-020, IRB-2014-05-001, and IRB-2014-05-002). Human blood samples were collected from 6 patients with r-axSpA who met the Assessment of SpondyloArthritis International Society classification criteria and 8 healthy controls. The characteristics of the human blood samples are given in Supplementary Table 1. peripheral blood monocytes (PBMCs) were separated from whole blood via Ficoll-Paque density gradient centrifugation (GE Healthcare, USA). Human bone tissues were obtained during surgery from the facet joints of 7 patients with r-axSpA and 7 patients with noninflammatory spinal disease as controls^33^. The characteristics of human facet joint samples are given in Supplementary Table 2. Primary bone-derived cells (BdCs) derived from facet joint tissues obtained during spinal surgery in AS patients were provided by Dr. Ye-Soo Park (Orthopedic Surgery, Guri Hospital, Hanyang University College of Medicine) and Dr. Sung Hoon Choi (Department of Orthopedic Surgery, Hanyang University Hospital). Human primary BdCs were cultured as reported previously^34^. In primary BdCs, matrix maturation during osteoblast differentiation was assessed via alkaline phosphatase (ALP; Sigma-Aldrich, St. Louis, MO, USA, 85L2) staining. The matrix mineralization of osteoblast differentiation was assessed using ARS (Sigma-Aldrich, A5533), hydroxyapatite HA (Lonza, PA-1503), and Von Kossa (Sigma-Aldrich, S7179) staining.

### Reagents

The following reagents were used in the in vitro experiments: losartan potassium (ARB; Sigma-Aldrich, SML3269), captopril (ACEi; Sigma-Aldrich, C4042), angiotensin II (Sigma-Aldrich, A6402), angiotensin 1-7 (R & D Systems, Minneapolis, MN, 1562), MLN-4760 (ACE2i; Sigma-Aldrich, 530616), sacubitrilat (NEPi; MedChem Express, Shanghai, China, HY-17620), bradykinin (R & D Systems, 3004), bradykinin receptor inhibitor (R & D Systems, HOE 140), and Mas receptor inhibitor (R & D Systems, A779).

### Osteoclast differentiation from mouse primary bone marrow-derived cells

Mouse bone marrow macrophages (BMMs), isolated by flushing the marrow space of the femur and tibia in 5-week-old mice, were incubated overnight on culture dishes in α-minimal essential medium (MEM) (Gibco Laboratories, Grand Island, NY, USA) containing 10% fetal bovine serum (FBS) (Gibco Laboratories) and antibiotics (100 units/mL penicillin G and 100 µg/mL streptomycin; Gibco Laboratories) at 37 °C in a humidified atmosphere containing 5% CO_2_ and 95% air. After adherent cells were discarded, the floating cells were further incubated with mouse macrophage colony stimulating factor (M-CSF) (30 ng/mL; Peprotech, USA) on Petri dishes. The BMMs became adherent after 3 days in culture, and the cells were differentiated into OCs after treatment with RANKL (50 ng/mL; Peprotech) and M-CSF (30 ng/mL; Peprotech) for 5 days.

### Osteoblast differentiation from mouse primary calvarial cells

ICR mice were purchased from DBL Co., Ltd. (South Korea). Murine calvarial cells were obtained from the calvaria of 1–3-day-old ICR mice and incubated for 15 min in α-MEM containing 0.1% collagenase (Gibco Laboratories) and 0.2% dispase (Gibco Laboratories) with shaking. The supernatant was collected and centrifuged for 5 min at 1 600 rpm. This step was repeated five times. Primary mouse calvarial cells were maintained in α-MEM with 10% FBS and antibiotics (100 units/mL penicillin G and 100 µg/mL streptomycin) at 37 °C in a humidified atmosphere containing 5% CO_2_ and 95% air and were differentiated into OBs following treatment with ascorbic acid (50 µg/mL; Sigma-Aldrich) and 10 mM β-glycerophosphate (Sigma-Aldrich) for 7–21 days.

### Tartrate-resistant acid phosphatase activity assay

After the BMMs were differentiated into OCs for 5 days, the cells were fixed with 4% formaldehyde for 15 min. Then, the cells were washed and stained for tartrate-resistant acid phosphatase (TRAP) using a TRAP staining kit (TaKaRa Biotechnology, Otsu, Japan) according to the manufacturer’s instructions. TRAP-positive multinucleated cells (MNCs) containing three or more nuclei were counted as mature OCs under a light microscope. All experiments were performed in triplicate with independent samples.

### Alkaline phosphatase staining and activity assay and matrix mineralization assay

After the cells were seeded in 24-well culture plates and differentiated for 7 and 21 days, they were fixed with 4% formaldehyde for 15 min. Then, the cells were washed, and ALP activity was determined using an ALP staining kit (TaKaRa Biotechnology) and an alkaline phosphatase yellow (pNPP) liquid substrate system (Sigma-Aldrich). For analysis of the matrix mineralization phase, alizarin red staining (ARS; Sigma-Aldrich) was used to determine calcium deposition. For ARS quantification, the stained wells were treated with 10% cetylpyridinium chloride (Sigma-Aldrich). The supernatant was transferred to a 96-well plate, and the absorbance was read at an excitation wavelength of 540 mM using an enzyme-linked immunosorbent assay (ELISA) plate reader.

### Assessment of osteoblast differentiation from human primary bone-derived cells

Matrix maturation during osteoblast differentiation was assessed via alkaline phosphatase (ALP; Sigma, 85L2) staining. The matrix mineralization of osteoblast differentiation was assessed via ARS (Sigma, A5533), hydroxyapatite HA (Lonza, PA-1503), and Von Kossa staining using 1% silver nitrate solution (Sigma, S7179). After staining, the wells were imaged using an Eclipse Ti-U microscope (Nikon, MEA510AA). For quantification via ARS staining, stained wells were incubated with 200 μL of 10% acetic acid at 37 °C for 2 h. Eighty microliters of the extracted solution was added to each well in new 96-well plates, and absorbance was measured at 450 nm using a multiplate reader (Thermo Scientific, Waltham, MA, USA, 51119000). For quantification of HA staining, the stained wells were measured at an excitation wavelength of 492 nm and an emission wavelength of 550 nm using a multiplate reader. For Von Kossa staining quantification, stained well images were selected and analyzed using ImageJ (National Institutions of Health, Bethesda, MD, USA).

### RNA isolation and reverse-transcription quantitative polymerase chain reaction (RT‒qPCR) analysis

Total RNA was extracted using TRIzol reagent (Molecular Research Center, Genbiotech SRL, Argentina) according to the manufacturer’s protocol. Reverse transcription was performed using ReverTra Ace qPCR RT master mix with a gDNA remover kit (Toyobo, Osaka, Japan) following the manufacturer’s instructions. Amplification of 1 µL of cDNA was performed using the iQ^TM^ SYBR Green Supermix (Bio-Rad Laboratories, Hercules, CA, USA). RT‒qPCR was performed using a CFX96 real-time PCR detection system (Bio-Rad Laboratories). The expression of mouse and human target genes was normalized to that of *18S rRNA* and *HuPo*, which were used as housekeeping genes. The normalized expression values were averaged, and the average fold changes were calculated. The detailed primer sequences are provided in Supplementary Table 3. Three independent experiments were performed to determine the mRNA levels.

### ELISA

The levels of angiotensin 1-7 and bradykinin in the supernatant collected from captopril (ACEi)-stimulated cells were determined using an ELISA kit (R & D Systems) according to the manufacturer’s protocol (NBP2-69079; Novus Biologicals, USA).

### Western blotting

Protein extracts were isolated from each group of cells using RIPA lysis buffer (Sigma-Aldrich) containing 1 mM complete protease inhibitor cocktail (Sigma-Aldrich). Total protein was separated using 10% sodium dodecyl sulfate‒polyacrylamide gel electrophoresis, transferred to a nitrocellulose membrane, blocked in 5% bovine serum albumin, probed with appropriate primary antibodies against the target proteins (anti-TRAF6, sc-8409, 1:200, Santa Cruz Biotechnology, Inc.; anti-β-actin, A1978, 1:5000, Sigma-Aldrich) overnight at 4 °C and then incubated with horseradish peroxidase-conjugated secondary antibodies (goat anti-mouse IgG H&L, ab6708, 1:10,000, Abcam; goat anti-rabbit IgG H&L, ab6721, 1:10,000, Abcam) at room temperature for 1 h. Protein bands were detected using a chemiluminescence reagent (Thermo Fisher Scientific).

### Cell Counting Kit-8 assay for cell viability

Cell viability was determined using the Cell Counting Kit-8 (CCK-8) assay. Briefly, the cells were plated in 96-well plates at a density of 1 × 10^4^ cells/well. After drug treatment, the cells were incubated for 4 h with CCK-8 reagent (Dojindo Molecular Technologies, Inc., Shanghai, China); then, absorbance was measured at 450 nm using a microplate reader.

### Statistical analysis

Statistical analysis was performed using GraphPad Prism 9 (GraphPad Software, San Diego, CA, USA). The Mann‒Whitney *U* test was performed for two-group comparisons, and data with *p* < 0.05 were considered statistically significant. All results are presented as the mean ± standard deviation (SD).

## Results

### Expression of RAS components was upregulated in the joints of SKGc mice and the PBMCs and BdCs of r-axSpA patients

**Fig. 1:**
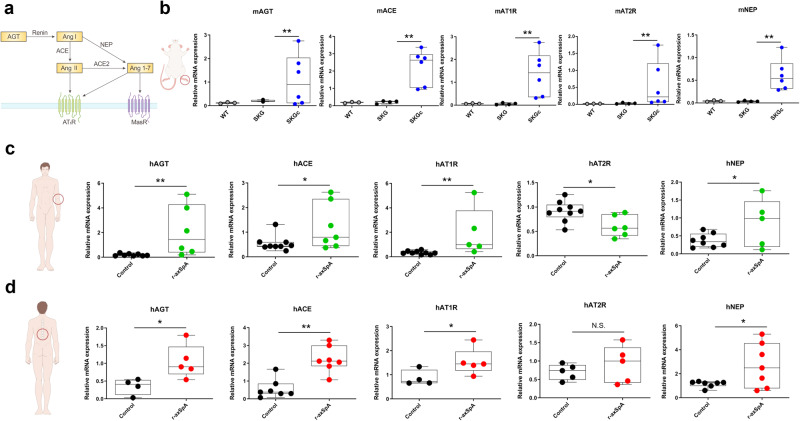
Expression levels of RAS components increased in SKGc mice and patients with r-axSpA. **a** Schematic representation of the renin-angiotensin system (RAS). **b**, **c** RT‒qPCR analysis of RAS component-related gene expression in (**b**) ankle joint tissues of wild-type (WT; *n* = 3), SKG (*n* = 3), and SKGc (*n* = 5) mice; **c** PBMCs of the controls (*n* = 8) and r-axSpA patients (*n* = 6); **d** bone-derived cells of the controls (*n* = 7) and r-axSpA patients (*n* = 7). ACE, angiotensin-converting enzyme; AGT, angiotensinogen; AT1/2 R, angiotensin II type 1/2 receptor; NEP, neutral endopeptidase; SKG, Sakaguchi; SKGc, curdlan-induced SKG; r-axSpA, radiographic axial spondyloarthritis; N.S., not significant. Values are presented as the mean ± S.D.; *p < 0.05, **p < 0.01 using the Mann‒Whitney *U* test.

To determine the expression of RAS components in the arthritic joints of an animal model of SpA, we injected SKG mice with curdlan to generate SKGc mice (Fig. 1a). The expression of RAS components in the joints of the SKGc mice was assessed using RT‒qPCR and Western blotting. The expression levels of AGT, ACE, AT1R, AT2R, and NEP were significantly higher in the joints of the SKGc mice than in those of the wild-type (WT) or SKG mice (*p* = 0.025, 0.034, 0.014, 0.020, and 0.011, respectively; Fig. 1b). In addition, Western blotting analysis results revealed that the protein levels of AGT, ACE, and AT1R in the ankle joints of the SKGc mice were higher than those in the WT or SKG mice (Supplementary Fig. 1).

We also compared the expression of RAS molecules between patients with r-axSpA and control participants. First, we identified the RAS molecules in the PBMCs of the patients with r-axSpA and the control participants. The expression levels of AGT, ACE, AT1R, AT2R, and NEP were significantly higher (*p* = 0.003, 0.0037, 0.005, 0.025, and 0.0306, respectively) in the PBMCs of the patients with r-axSpA than in those of the control participants (Fig. 1c). Next, we compared the expression of RAS molecules in BdCs between the patients with r-axSpA and the control participants. The expression levels of all RAS molecules, except for AT2R, were significantly higher (*p* = 0.027, 0.003, 0.05, and 0.032) in the r-axSpA patients than in the control participants (Fig. 1d).

### ARBs, but not ACEis, inhibited bone erosion and systemic bone loss in SKGc mice

**Fig. 2:**
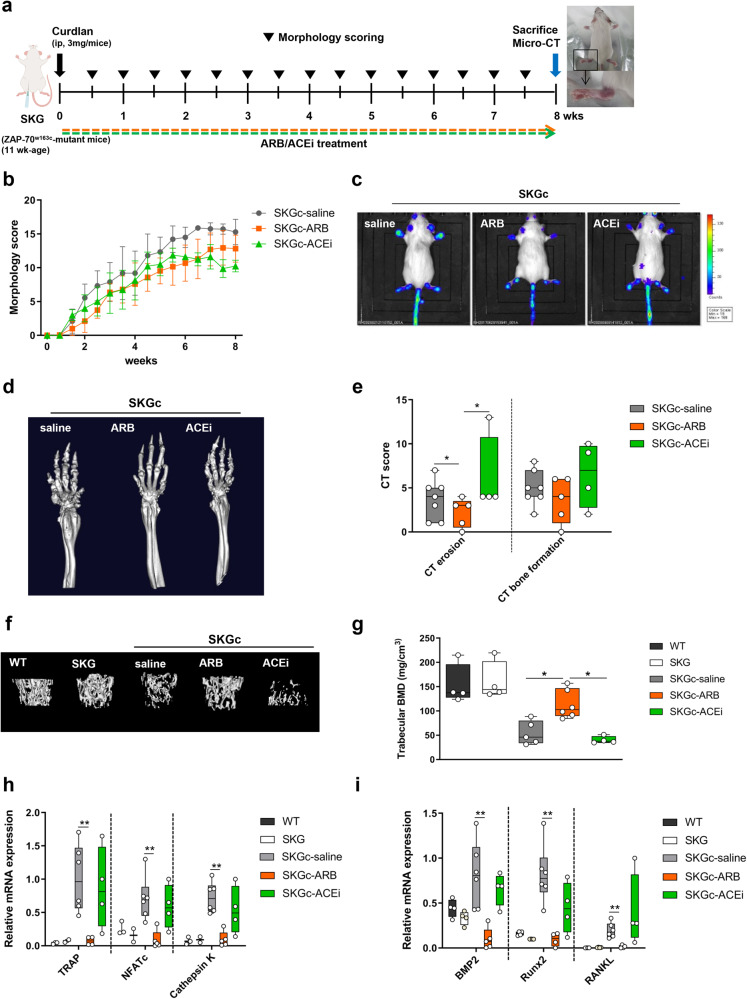
ARBs and ACEis differentially affected bony changes but did not affect clinical arthritis in SKGc mice. **a** Experimental design. Arthritis was induced in SKG mice by injecting 3 mg curdlan (SKGc), and samples were acquired at 8 weeks. **b**–**i** SKGc mice treated with saline, ARB (10 mg/kg losartan), or ACEi (10 mg/kg enalapril). **b** Morphological scoring. **c** MPO activity measured using IVIS. **d** Micro-CT images of ankle joints. **e** CT scoring of bone erosion and formation. **f** Micro-CT images of trabecular bones. **g** Measurement of BMD. mRNA expression of osteoclast (**h**) and osteoblast (**i**) differentiation markers in ankle joints (*n* = 5 for group). The 18S RNA expression was used for normalization. ACEi, angiotensin-converting enzyme inhibitor; ARB, angiotensin II receptor blocker; SKG, Sakaguchi; SKGc, curdlan-induced SKG. Values are presented as the mean ± S.D.; *p < 0.05, **p < 0.01 using the Mann‒Whitney *U* test.

RAS affects the development of inflammation^35^. Hence, we investigated whether RAS modulators, such as ARBs and ACEis, influence the development of arthritis in SKGc mice. First, we monitored the severity of paw swelling in each limb and compared the clinical arthritis scores among the SKGc groups treated with vehicle, ARB, or ACEi (SKGc-saline, SKGc-ARB, or SKGc-ACEi, respectively) (Fig. 2a). No significant differences were observed between the groups (Fig. 2b). Next, we measured MPO activity using IVIS; however, we did not find any differences between the groups (Fig. 2c).

In addition, joint tissues were stained with hematoxylin and eosin to determine the degree of inflammatory cell infiltration in the ankle joints of the SKGc mice. Compared with the WT mice, the SKGc-saline mice showed increased inflammatory cell infiltration in the joints. There was no difference in the degree of inflammatory cell infiltration among the SKGc-saline, SKGc-ARB, and SKGc-ACEi mice, which is consistent with the clinical arthritis score and MPO activity (Supplementary Fig. 2). Thus, RAS modulators did not significantly affect the severity of arthritis in the SKGc mice.

Subsequently, we performed CT of arthritic joints to assess the effect of RAS modulation on the development of erosion and abnormal bone formation. The CT erosion score was significantly lower in the SKGc-ARB mice (*p* = 0.033) and higher in the SKGc-ACEi mice than in the SKGc-saline mice (*p* = 0.898) (Fig. 2d, e, left). CT bone formation scores decreased in the SKGc-ARB mice (*p* = 0.515) and increased in the SKGc-ACEi mice (*p* = 0.516) compared to those in the SKGc-saline mice (Fig. 2d, e, right).

Furthermore, bone CT was used to investigate the effect of RAS modulation on bone remodeling. Bone mineral density (BMD) was significantly higher in the SKGc-ARB group (*p* = 0.011) and lower in the SKGc-ACEi group (*p* = 0.462) than in the SKGc-saline group (Fig. 2f, g). Differences were not observed in BMD among the WT-saline, WT-ARB, and WT-ACEi groups treated with each RAS modulator (to determine whether these drugs affected BMD in a noninflammatory environment) (Supplementary Fig. 3a, b).

Next, we measured the expression levels of bone cell-related molecules in the joints of SKGc mice. The expression levels of OC differentiation markers, including TRAP, NFATc, and cathepsin K, were significantly lower in the SKGc-ARB mice than in the SKGc-saline mice (*p* = 0.006, 0.006, and 0.006, respectively); however, differences were not observed between the SKGc-ACEi and SKGc-saline mice (*p* = 0.522, 0.670, and 0.394, respectively) (Fig. 2h). Similarly, the expression levels of OB differentiation markers, including BMP2, RUNX2, and RANKL, were significantly lower in the SKGc-ARB mice (*p* = 0.006, 0.006, and 0.006, respectively) but higher in the SKGc-ACEi mice than in the SKGc-saline mice (*p* = 0.286, 0.088, and 0.394, respectively) (Fig. 2i).

### ARBs and ACEis showed opposite effects on osteoclast differentiation of mouse primary bone marrow macrophages

**Fig. 3:**
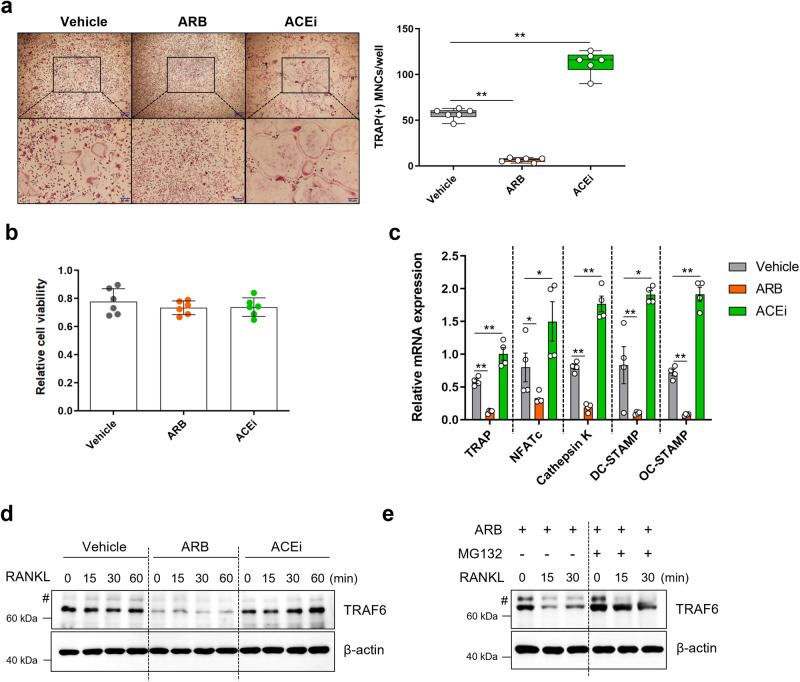
ARBs inhibited and ACEis promoted osteoclast differentiation in mouse cells. **a**–**c** Mouse bone marrow monocytes were treated with ARB (1 × 10^−5^ M losartan) or ACEi (1 × 10^−5^ M captopril). **a** Representative TRAP staining images (left) and the number of TRAP-positive MNCs (>3 nuclei) per well (right). Scale bar = 50 µm. **b** CCK-8 assay. **c** RT‒qPCR analysis. **d** Western blotting analysis of ARB- or ACEi-treated RAW 264.7 cells. **e** Western blotting analysis of ARB-treated RAW 264.7 cells with/without MG132 treatment. The hash symbol (#) indicates nonspecific bands. ACEi, angiotensin-converting enzyme inhibitor; ARB, angiotensin II receptor blocker; MNCs, multinuclear cells; TRAP, tartrate-resistant acid phosphatase. Values are presented as the mean ± S.D.; *p < 0.05, **p < 0.01 using the Mann‒Whitney *U* test.

To verify the direct role of RAS in bone cells, we applied RAS modulators in bone cell culture systems. First, we administered ARBs or ACEis to the BMMs of mice. TRAP staining revealed that OC differentiation was significantly inhibited by ARBs (*p* = 0.006) but significantly promoted by ACEis (*p* = 0.004) compared to that in the controls (Fig. 3a). ACEis increased the numbers of TRAP-positive multinucleated OCs and OCs with more than 30 nuclei per cell (*p* = 0.002; Supplementary Fig. 4). Differences in cell viability were not observed among the groups in the CCK-8 assay (Fig. 3b). RT‒qPCR analysis revealed that ARB significantly lowered (*p* = 0.006, 0.028, 0.009, 0.009, and 0.009, respectively) whereas ACEi increased (*p* = 0.006, 0.028, 0.009, 0.021, and 0.009, respectively) the expression levels of OC differentiation markers, including TRAP, NFATc, cathepsin K, DC-STAMP, and OC-STAMP, compared with those in the controls (Fig. 3c).

To investigate the signals acting upstream of the abovementioned factors, we measured the expression level of TRAF6 in RAW 264.7 cells treated with saline, ARBs, or ACEis. ARBs inhibited the expression of TRAF6, but ACEis did not (Fig. 3d). To further investigate the mechanism underlying the inhibition, we treated cells with MG132, a proteasome inhibitor, which restored TRAF6 expression (Fig. 3e), suggesting that the effect of RAS modulators on OCs was mediated via TRAF6 ubiquitination, resulting in degradation by the proteasome.

### Ang 1-7 facilitated osteoclast differentiation from mouse primary bone marrow macrophages

**Fig. 4:**
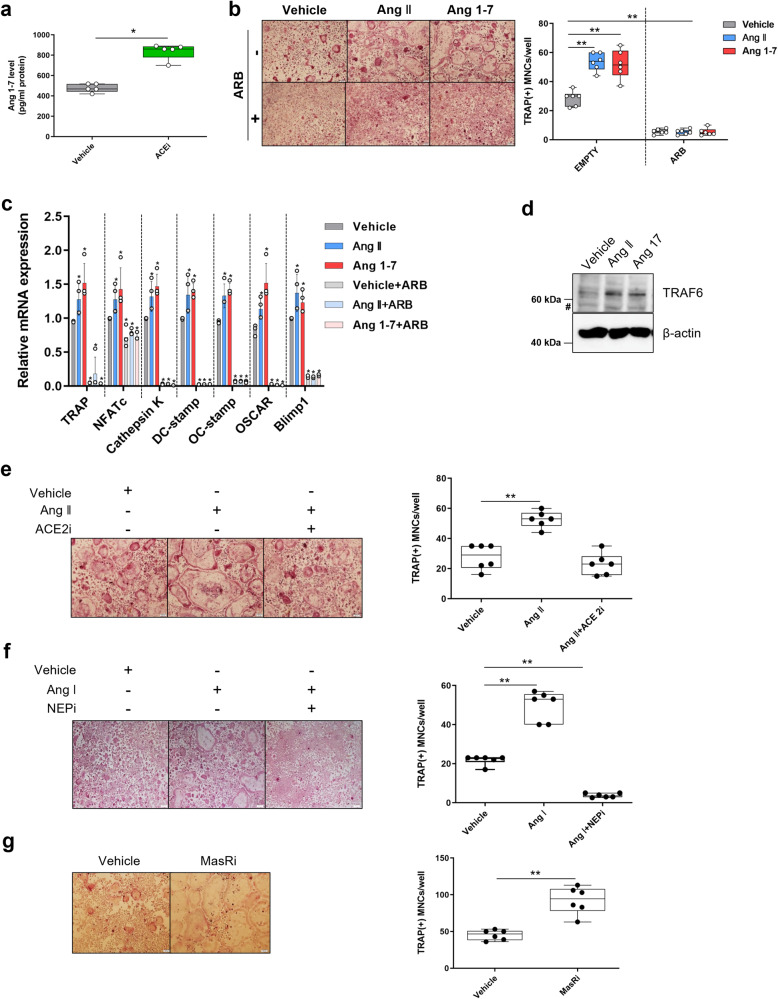
Ang 1-7 promoted osteoclast differentiation in mouse cells. **a** Level of Ang 1-7 measured using ELISAs in osteoclast culture media with/without ACEi (1 × 10^−5^ M captopril). **b**, **c** Mouse bone marrow monocytes were treated with Ang II (1 × 10^−6^ M) or Ang 1-7 (1 × 10^−6^ M) with/without ARB (1 × 10^−5^ M losartan). **b** Representative TRAP staining images (left) and the number of TRAP-positive MNCs (>3 nuclei) per well (right). **c** RT‒qPCR analysis. **d** Western blotting analysis of Ang II- or Ang 1-7-treated RAW 264.7 cells. Hash indicates nonspecific bands. **e**–**g** Representative TRAP staining images (left) and the number of TRAP-positive MNCs (>3 nuclei) per well (right) before and after Ang II (1 × 10^−6^ M) administration with/without ACE2i (1 × 10^−5^ M MLN-4760) (**e**), Ang I (1 × 10^−6^ M) with/without NEPi (1 × 10^−5^ M sacubitrilat) (**f**), and Ang I with/without MasRi (1 × 10^−5^ M A779) (**g**). Scale bar = 50 µm. ACE2i, angiotensin-converting enzyme 2 inhibitor; Ang, angiotensin; MasRi, Mas receptor inhibitor; MNCs, multinuclear cells; NEPi, neprilysin inhibitor; TRAP, tartrate-resistant acid phosphatase. Values are presented as the mean ± S.D.; *p < 0.05, **p < 0.01 using the Mann‒Whitney *U* test.

The conflicting effects of ARBs and ACEis led us to hypothesize that contrary to previous reports^22,36,37^, Ang 1-7, rather than Ang II, might play a dominant role in OC differentiation. To verify this, we first confirmed that Ang 1-7 levels increased significantly (*p* = 0.021) after OC progenitors were treated with ACEis (Fig. 4a).

To examine the effect of Ang 1-7 on OC differentiation, we treated the cells with saline, Ang 1-7, or Ang II. TRAP staining revealed that Ang 1-7 enhanced OC differentiation (*p* = 0.004; Fig. 4b) and the expression of target molecules, including TRAP, NFATc, cathepsin K, DC-STAMP, OC-STAMP, OSCAR, and Blimp1 (*p* = 0.020, 0.018, 0.017, 0.019, 0.017, 0.019, and 0.019, respectively), to an extent similar to that observed with Ang II (Fig. 4c). Both Ang II and Ang 1-7 increased the expression of TRAF6, contrary to that observed with ARBs (Fig. 4d).

We hypothesized that the increase in OC differentiation after treatment with Ang II was caused by Ang 1-7 derived from Ang II and not by Ang II itself. Therefore, we simultaneously administered Ang II and an ACE2i to OC progenitor cells to inhibit conversion. This treatment diminished the promoting effect of Ang II on OC differentiation to a level similar to that of the control group (Fig. 4e). Furthermore, Ang 1-7 levels decreased when an ACE2i was used in combination with Ang II compared to Ang II alone (Supplementary Fig. 5). In addition, an NEP inhibitor (NEPi), administered with Ang I, completely blocked OC differentiation (*p* = 0.002; Fig. 4f), implying that NEP might be a key enzyme involved in Ang 1-7 production, at least in the bone milieu. OC differentiation was inhibited by NEPi treatment (*p* = 0.004) without changes in cell viability (Supplementary Fig. 6a, b), and the mRNA expression of target molecules, including TRAP, NFATc, cathepsin K, DC-STAMP, and OC-STAMP, decreased after NEPi treatment (*p* = 0.008, 0.008, 0.008, 0.008, and 0.009, respectively; Supplementary Fig. 6c). These results, combined with the finding that ACEis promoted OC differentiation, suggested that Ang 1-7, rather than Ang II, plays a major role in OC differentiation.

Furthermore, a Mas receptor (MasR) inhibitor (MasRi) was used to assess OC differentiation, as MasR is the major receptor of Ang 1-7. MasRi treatment significantly increased OC differentiation (*p* = 0.009; Fig. 4g). In addition, to determine whether Ang 1-7 acted on the AT1 receptor, we simultaneously treated OC progenitor cells with Ang 1-7 and ARB, which suppressed the Ang 1-7-induced promotion of OC differentiation (Fig. 4b, c). To further verify this finding, we simultaneously administered ACEis and ARBs to the cells. We observed that OC differentiation was inhibited without changes in cell viability (Supplementary Fig. 7a, b), and expression of the OC differentiation marker decreased (Supplementary Fig. 7c). Therefore, the Ang 1-7/AT1 axis is the major pathway involved in OC differentiation.

### ARBs and ACEis exerted opposite effects on osteoblast differentiation from mouse primary osteoblast progenitor cells

**Fig. 5:**
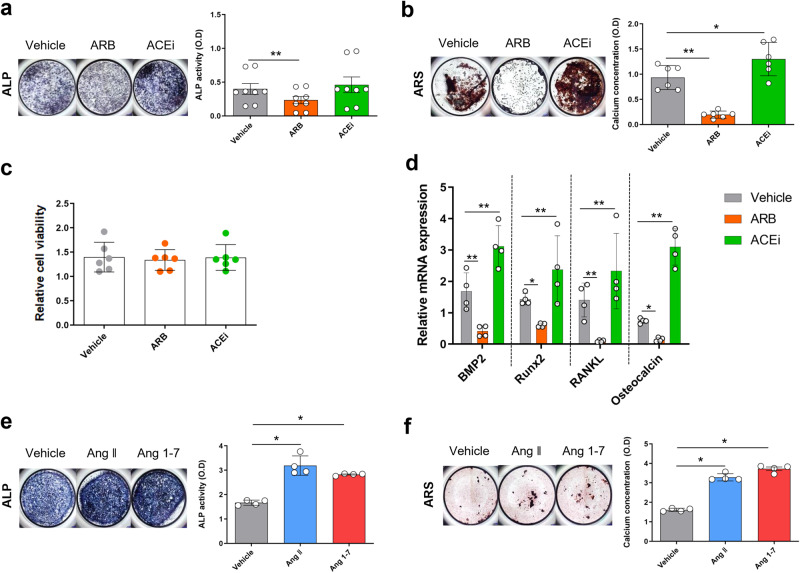
ARBs, but not ACEis, inhibited osteoblast differentiation in mouse cells. **a**–**d** ARB (1 × 10^−5^ M losartan) or ACEi (1 × 10^−5^ M captopril) administered to primary mouse osteoblastic cells. **a** ALP staining (left) and ALP activity (right). **b** ARS staining (left) and its quantification (right). **c** CCK-8 assay. **d** RT‒qPCR analysis. **e**, **f** Ang II (1 × 10^−6^ M) or Ang 1-7 (1 × 10^−6^ M) was applied to primary mouse osteoblastic cells. **e** ALP staining (left) and ALP activity (right). **f** ARS staining (left) and its quantification (right). ACEi, angiotensin-converting enzyme inhibitor; ALP, alkaline phosphatase; Ang, angiotensin; ARB, angiotensin II receptor blocker; ARS, alizarin red. Values are presented as the mean ± S.D.; *p < 0.05, **p < 0.01 using the Mann‒Whitney *U* test.

To examine the effect of ARBs and ACEis on OB differentiation, we treated mouse OB progenitor cells with RAS modulators in an in vitro culture system. Intracellular ALP activity assays and alizarin red staining (ARS) were performed to assess OB differentiation and mineralization, respectively. Intracellular ALP activity in OBs was suppressed by ARB treatment (*p* = 0.021) but promoted by ACEi treatment (*p* = 0.11), suggesting that ARBs and ACEis exerted opposite effects on OB differentiation despite the lack of statistical significance (Fig. 5a). ARS analysis revealed that ARBs significantly inhibited (*p* = 0.021), whereas ACEis promoted (*p* = 0.043) mineralization (Fig. 5b) without changes in cell viability (Fig. 5c). Consistent with these findings, we observed that when compared with vehicle, ARBs significantly decreased the expression of bone formation markers, including BMP2, RUNX2, RANKL, and osteocalcin, in OBs (*p* = 0.021, 0.020, 0.021, and 0.021, respectively), whereas ACEis significantly increased their expression levels (*p* = 0.043, 0.248, 0.149, and 0.021, respectively) (Fig. 5d). Experiments using the human SaOS2 cell line yielded similar results (Supplementary Fig. 8a, b).

In addition, Ang 1-7 increased OB differentiation and mineralization to a degree similar to that of Ang II (Fig. 5e, f). When cells were costimulated by ARBs and ACEis, OB differentiation and mineralization were inhibited without changes in cell viability (Supplementary Fig. 9a–c).

### ARBs inhibited osteoblast differentiation from BdCs of r-axSpA patients

**Fig. 6:**
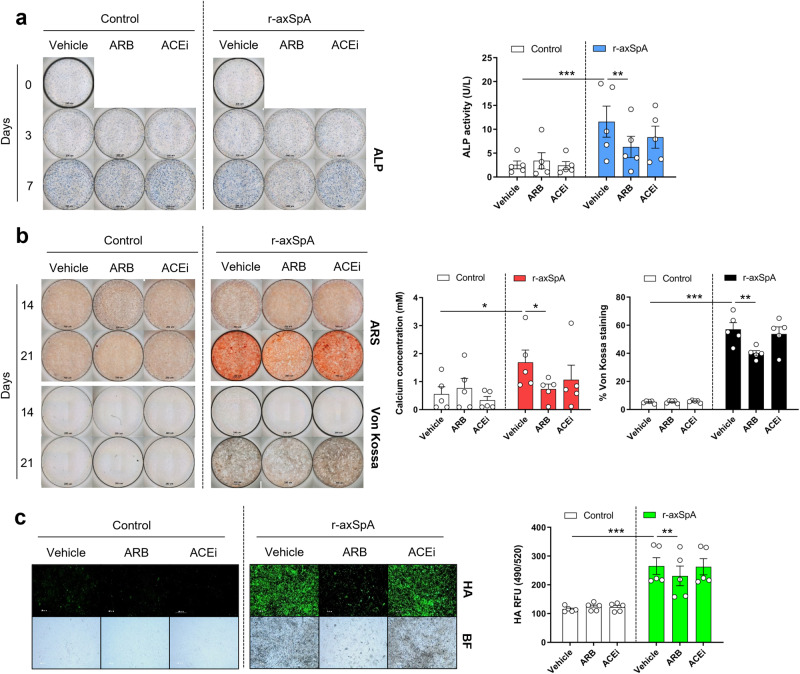
ARB suppressed osteoblast differentiation from BdCs of r-axSpA patients. **a**–**c** BdCs of the controls and r-axSpA patients were differentiated into osteoblasts using an ARB (1 × 10^−5^ M losartan) or ACEi (1 × 10^−5^ M captopril). **a** ALP staining (left) and ALP activity (right). **b** ARS and Von Kossa staining (left) and quantification (right). **c** HA staining (left) and quantification (right). Scale bar = 200 µm. ACEi, angiotensin-converting enzyme inhibitor; ALP, alkaline phosphatase; Ang, angiotensin; ARB, angiotensin II receptor blocker; ARS, alizarin red; AT1R, angiotensin II type 1 receptor; BdCs, bone-derived cells; HA, hydroxyapatite. Values are presented as the mean ± S.D.; *p < 0.05, **p < 0.01, ***p < 0.001 using the Mann‒Whitney *U* test.

We investigated whether RAS modulators affect the differentiation of OBs obtained from patients with r-axSpA. ARBs and ACEis were administered to the BdCs of biologic-naive r-axSpA patients and control participants. ALP, ARS, and Von Kossa staining revealed that the ARB significantly inhibited OB differentiation and mineralization in the biologic-naive r-axSpA patients (*p* = 0.0058, 0.042, 0.003, and 0.0144, respectively), whereas the ACEi did not (Fig. 6a, b, right panels). Significant changes were not observed in the OBs of the control participants treated with the ARB or ACEi (Fig. 6a, b, left panels). In addition, HA staining and bright field imaging showed that the ARB inhibited the mineralization of OBs in the r-axSpA patients but not in the control participants (Fig. 6c).

## Discussion

**Fig. 7:**
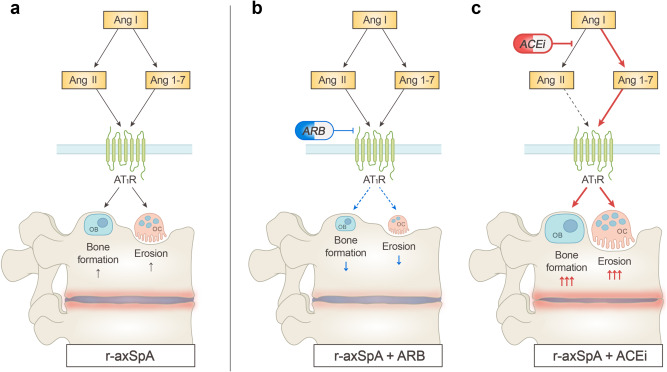
Schematic model showing the role of RAS inhibitors in SpA-associated bone changes. **a** Osteoclast and osteoblast differentiation increased in r-axSpA, possibly because of the increase in the expression of RAS molecules, culminating in aberrant bone formation and erosion. **b** An ARB inhibited the binding of Ang II and Ang 1-7 to AT1R, resulting in the suppression of osteoclast and osteoblast differentiation. **c** An ACEi inhibited the conversion of Ang I into Ang II and augmented the production of Ang 1-7, leading to increased osteoblast and osteoclast differentiation, which caused higher bone formation and erosion. Ang, angiotensin; ACEi, angiotensin-converting enzyme inhibitor; ARB, angiotensin II receptor blocker; AT1R, angiotensin II type 1 receptor; RAS, renin-angiotensin system; r-axSpA, radiographic axial spondyloarthritis.

Our findings indicated that RAS is involved in the differentiation of OBs and OCs and that RAS components are overexpressed in patients with r-axSpA. To the best of our knowledge, this is the first study to demonstrate the role of RAS in the pathogenesis of SpA. As RAS expression is upregulated in the PBMCs and bone cells of patients with r-axSpA (Fig. 7a), its modulation may effectively inhibit abnormal bone changes in patients with SpA. The effect of RAS modulators, however, depends on their type (Fig. 7b, c).

There are several plausible mechanisms by which systemic RAS may affect bone changes. RAS regulates the immune system^35^, which can indirectly affect bone remodeling, and interacts with calcium-regulating hormones^38,39^. However, we did not observe any significant differences in clinical arthritis scores, MPO activity, or inflammatory cell infiltration among the control, ARB-treated, or ACEi-treated groups. Thus, the bone changes modulated by ARBs and ACEis in SpA were not due to their effect on inflammation. Therefore, we performed in vitro studies to verify the direct effects of RAS on bone cells. Surprisingly, ARB and ACEi exerted different effects in animal models and in vitro culture systems, which is supported by the results of other studies on RAS modulators and osteoporosis. For example, treatment with the ARB olmesartan significantly reduced the decline in femoral neck BMD in bedridden elderly female hypertension patients with disuse syndrome^40^. Kwok et al. showed that ACEi use correlated with a small but significant increase in bone loss at the hip, whereas ARB use did not^41^. Another study conducted in Taiwan reported an increase in osteoporotic fractures with ACEi use but not with ARB use^42^.

However, whether RAS modulators affect osteoporosis development in patients receiving these medications remains unclear^43^, as double-blind placebo-controlled prospective studies have not yet been conducted. Generally, individuals receiving hypertensive medication are older and at risk of developing osteoporosis. In our study, ARB significantly inhibited systemic bone loss in SKGc mice, which can be explained by the high expression of RAS molecules presumed to cause bone loss. However, RAS modulators may not exert a significant effect on BMD in the general population in which the expression of RAS molecules is low, as ARB did not affect BMD in WT mice. Corroborating this claim, a recent study demonstrated that Ang II enhances bone erosion in a mouse model of RA but does not induce systemic bone loss in WT mice^28^. As r-axSpA patients showed higher expression of RAS components than control participants, ARBs could inhibit osteoporosis in patients with r-axSpA. The mechanisms underlying the increase in the expression of RAS components warrant further clarification.

We found that ARBs inhibited abnormal bone formation, increased BMD, and decreased OB and OC differentiation, whereas ACEis did not affect abnormal bone formation or BMD but increased OB and OC differentiation. Although Ang II is the main effector in bone metabolism^22,36,37^, our findings suggest that Ang 1-7 might be another important factor, as ACEis increased both OC and OB differentiation. ACEi increased the concentration of Ang 1-7 by inhibiting the conversion of Ang I to Ang II, thereby allowing the conversion of Ang 1 to Ang 1-7 and simultaneously inhibiting the degradation of Ang 1-7 to inactive Ang 1-5. Our in vitro studies with Ang 1-7 and enzyme inhibitors, including ACE2i and NEPi, indicated that Ang 1-7 might be an active mediator of bone metabolism. Nozato et al. reported that Ang 1-7 significantly increased bone volume in WT and *Ace2*-knockout mice^44^, which supports our hypothesis. Recently, Sha et al. reported that Ang 1-7 increased the formation of calcific nodules and ALP activity in OB cells, as well as bone mineralization, and decreased osteoclast differentiation via MasR in the presence of high levels of glucose^45^. These findings are consistent with our observation regarding OBs but not OCs. This discrepancy may be attributed to the hyperglycemic conditions in the previous study. We also postulate that Ang 1-7 may act on both MasR and AT1R and exert different effects depending on the receptor it binds to. The effect of Ang II on osteoclastogenesis has been reported to be exerted via Ang 1-7 conversion^16^. However, further studies are required to decipher the precise roles of Ang II and Ang 1-7 in bone cells.

Moreover, our in vitro observations indicate that in addition to RANKL signaling, RAS signaling is integral to the activation of TRAF6, a molecule acting upstream in osteoclastogenesis. ACEis did not increase TRAF6 expression but increased NFATc expression at the mRNA level, suggesting that additional downstream signals may be activated by ACEis.

Nevertheless, the present study had some limitations. First, although Ang 1-7 acts on AT1R^46^, we did not directly investigate whether Ang 1-7 binds to AT1R in OBs and OCs. As Ang II has a higher affinity for AT1R, the interaction between Ang II and Ang 1-7 may differentially affect bone cell differentiation. Further studies are required to provide compelling evidence regarding this issue. Second, factors other than Ang 1-7 may explain the differences observed between ARBs and ACEis. For example, in our study, bradykinin and Ang 1-7 promoted OC differentiation (*p* = 0.002 and 0.002, respectively; Supplementary Fig. 10). However, the effect was not inhibited by a bradykinin inhibitor (Supplementary Fig. 11). In addition, bradykinin levels did not differ significantly between the ACEi-treated and control groups (Supplementary Fig. 12), suggesting that the ACEi exerts a minimal effect on bradykinin degradation in the bone milieu. In contrast, Ang 1-7 levels increased significantly in the ACEi-treated group, indicating that Ang 1-7 was a more likely candidate than bradykinin in promoting OC differentiation. Third, the precise mechanisms by which Ang 1-7 enhances OB differentiation remain unclear. Last, given the small sample size and lack of power calculation, it is difficult to generalize the findings and conclude that regulating RAS reflects an imbalance in osteoblasts and osteoclasts in pathological bone changes in spondyloarthritis. Thus, these findings need to be validated in a large and multicenter cohort study.

This study has several important clinical implications. This is possibly the first study to suggest that RAS and its modulation may affect bone phenotype in SpA. The RAS modulator ARB significantly reduced bone erosion and increased BMD in an animal model of SpA, implying that ARBs may be better than ACEis for treating hypertension in patients with SpA. Our in vitro results also suggest that Ang 1-7 may play an important role in the differentiation of bone cells.

In conclusion, the RAS plays a key role in OC and OB differentiation, ARBs and ACEis exert different effects on abnormal bone changes in SpA, and Ang 1-7 may be a major effector of bone cell differentiation. In addition, RAS is a potential therapeutic target in SpA.

### Supplementary information

Supplementary Information

## Data Availability

The data used to support the findings of this study are included within the article and Supplementary material. Raw data that support the findings of this study are available from the corresponding author upon reasonable request.
