# Supplementary material for: Angiotensin receptor blockers, but not angiotensin-converting enzyme inhibitors, inhibit abnormal bone changes in spondyloarthritis
Source: Exp Mol Med. 2023 Nov 1;55(11):2346–56. doi: 10.1038/s12276-023-01103-z (PMC10689434; doi:10.1038/s12276-023-01103-z)
Supplement: Supplementary file 1 — Supplementary Information [file 12276_2023_1103_MOESM1_ESM.pdf]

Angiotensin receptor blockers, but not angiotensin-converting enzyme inhibitors, inhibit abnormal bone changes in spondyloarthritis

This PDF file includes:

Supplementary Materials and Methods

Supplementary Figures

Supplementary Tables

Supplementary Materials and Methods

Reagents

The following reagents were used in the in vitro experiments: sacubitrilat (NEPi; MedChem Express, Shanghai, China, HY-17620), bradykinin (R & D Systems, Minneapolis, MN, 3004), and bradykinin receptor inhibitor (R & D Systems, HOE 140).

Histological analysis

The mice were euthanized on day 56 and their hind ankle joints were dissected. Mouse joint tissues were fixed in 10% formalin, decalcified for 3 days in 10% EDTA, and embedded in paraffin wax blocks. Five-micrometer sections were stained with hematoxylin and eosin.

In vitro **cell line and osteoclast differentiation**

The RAW264.7 monocyte/macrophage cell line was provided by Professor Tae-Hwan Kim (Department of Rheumatology, Hanyang University Hospital for Rheumatic Diseases) and maintained in Dulbecco′s Modified Eagle′s Medium (DMEM; Gibco Laboratories, Grand Island, NY, USA) supplemented with 10% FBS (Gibco Laboratories) and antibiotics (100 units/mL penicillin G and 100 μg/mL streptomycin; Gibco Laboratories) at 37 °C in a humidified atmosphere containing 5% CO_2 and 95% air. The RAW264.7 cells were seeded onto the prepared matrix in a 35-mm dish. After 2 days, the cells were differentiated into OCs using RANKL (50 ng/mL; Peprotech).

In vitro **cell line and osteoblast differentiation**

The osteosarcoma cell line SaOS2 was generously provided by professor Tae-Hwan Kim (Department of Rheumatology, Hanyang University Hospital for Rheumatic Diseases). The SaOS2 cell line was grown in high-glucose DMEM (Gibco Laboratories) with 10% FBS and antibiotics (100 units/ml penicillin G and 100 μg/ml streptomycin) at 37 °C in a humidified

atmosphere containing 5% CO_2 and 95% air, and was differentiated into OBs using 50 μM ascorbic acid (Sigma–Aldrich, USA), 10 mM β-glycerophosphate (Sigma–Aldrich), and 100 nM dexamethasone (Sigma–Aldrich) for 7–14 days.

Western blotting

Protein extracts were isolated from each group of cells using RIPA lysis buffer (Sigma-Aldrich) containing 1 mM complete protease inhibitor cocktail (Sigma-Aldrich). Total protein was separated using 10% sodium dodecyl sulfate-polyacrylamide gel electrophoresis , transferred to a nitrocellulose membrane, blocked in 5% bovine serum albumin, probed with appropriate primary antibodies against the target proteins (anti-AGT, ab97381, 1:500, Abcam, Cambridge, UK; anti-ACE, ab28311, 1:1000, Abcam; anti-AGTR1, ab124734, 1:1000, Abcam; anti-β-Actin, A1978, 1:5000, Sigma–Aldrich) overnight at 4 °C, and then incubated with horseradish peroxidase-conjugated secondary antibodies (goat anti-mouse IgG H&L, ab6708, 1:10000, Abcam; goat anti-rabbit IgG H&L, ab6721, 1:10000, Abcam) at room temperature for 1 h. Protein bands were detected using a chemiluminescence reagent (Thermo Fisher Scientific).

Supplementary Figures

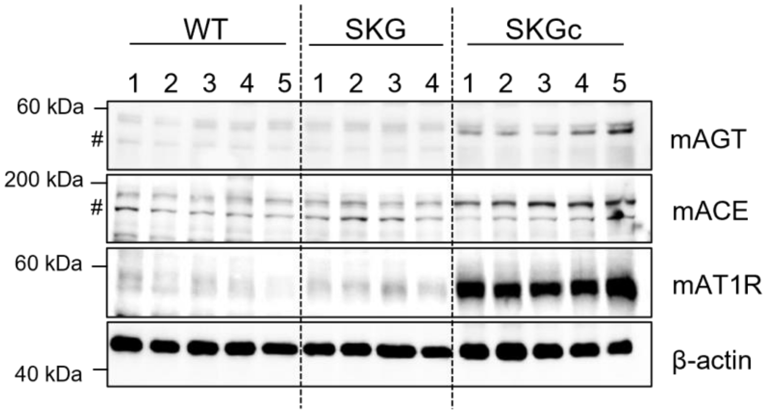

Supplementary Fig. 1. Expression levels of RAS components increased in the ankle joints of SKGc mice. Arthritis was induced in SKG mice for 8 weeks after injecting 3 mg curdlan. Ankle joint tissues of WT (n = 5), SKG (n = 4), and SKGc (n = 5) mice were ground, and lysates were obtained. Western blotting of ankle joint lysates using mAGT, mACE, mAT1R, and β-actin antibodies. Hash symbol (#) indicates nonspecific bands. mACE, mouse angiotensin-converting enzyme; mAGT, mouse angiotensinogen; mAT1R, mouse angiotensin II type 1 receptor; SKG, Sakaguchi; SKGc, curdlan-induced SKG.

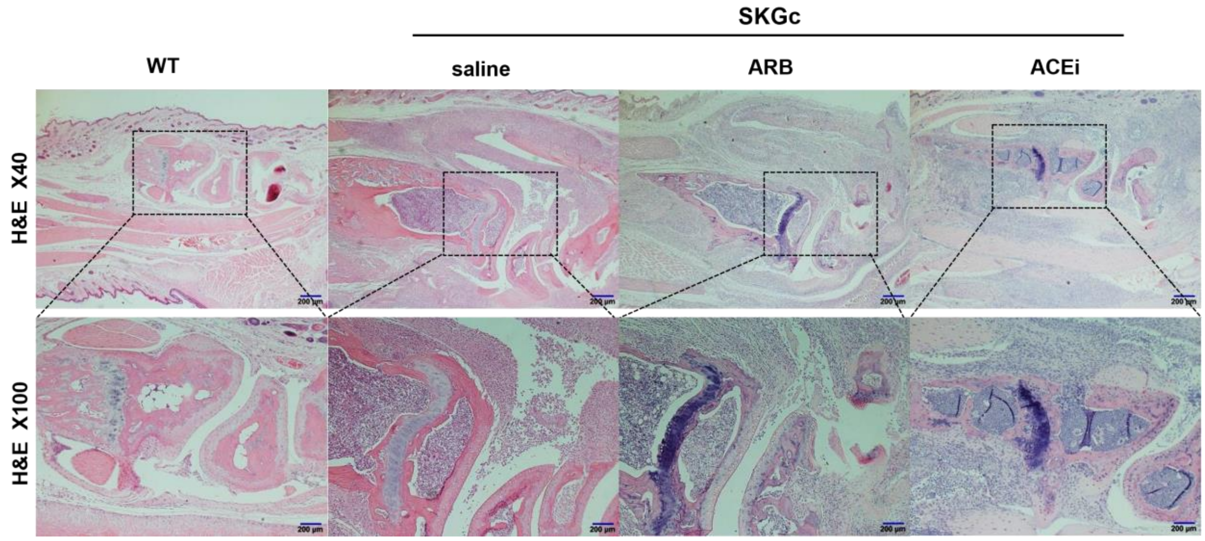

Supplementary Fig. 2. ARB and ACEi did not affect the severity of clinical arthritis in SKGc mice. Saline, ARB (10 mg/kg losartan), or ACEi (10 mg/kg enalapril) was administered to SKG mice for 8 weeks after injecting 3 mg curdlan. Mouse joint tissues were dissected, fixed in 10% formalin, decalcified for 3 days in 10% EDTA, and embedded in paraffin wax blocks. Five-micrometer sections were stained with hematoxylin and eosin (H & E). Scale bar = 200 µm. ACEi, angiotensin-converting enzyme inhibitor; ARB, angiotensin II receptor blocker; SKG, Sakaguchi; SKGc, curdlan-induced SKG.

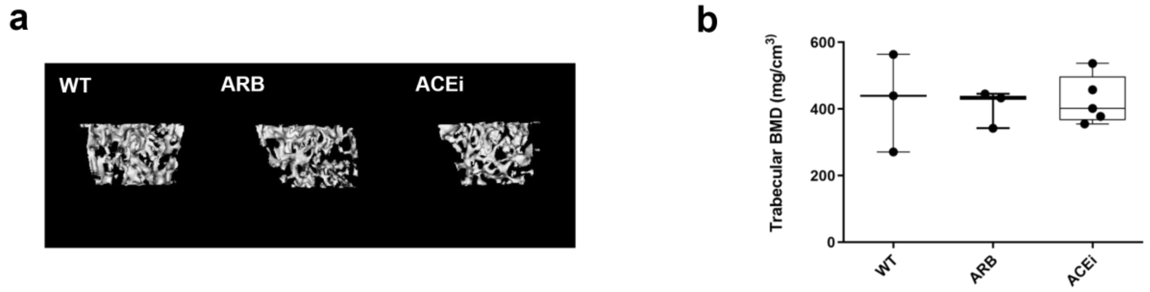

Supplementary Fig. 3. ARB and ACEi did not affect BMD in mice in the absence of inflammation. (a) Representative CT images of lumbar vertebra and (b) trabecular BMD of BALB/c mice treated with ARB (10 mg/kg losartan) or ACEi (10 mg/kg enalapril). ACEi, angiotensin-converting enzyme inhibitor; ARB, angiotensin II receptor blocker; BMD, bone mineral density.

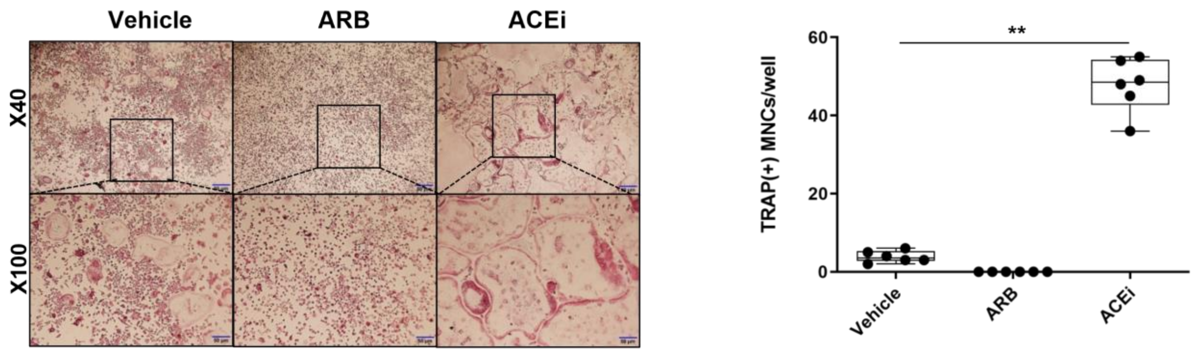

Supplementary Fig. 4. ARB inhibited, while ACEi promoted osteoclast differentiation. Mouse bone marrow monocytes were treated with ARB (1×10^{-5} M losartan) or ACEi (1×10^{-5} M captopril). Representative TRAP staining images (left) and the number of TRAP-positive MNCs (>30 nuclei) per well (right). Scale bar = 50 µm. ACEi, angiotensin-converting enzyme inhibitor; ARB, angiotensin II receptor blocker; MNCs, multi-nuclear cells; TRAP, tartrate-resistant acid phosphatase. Data are presented as the mean ± SD.; $^{**}p < 0.01$ using Mann-Whitney *U* test.

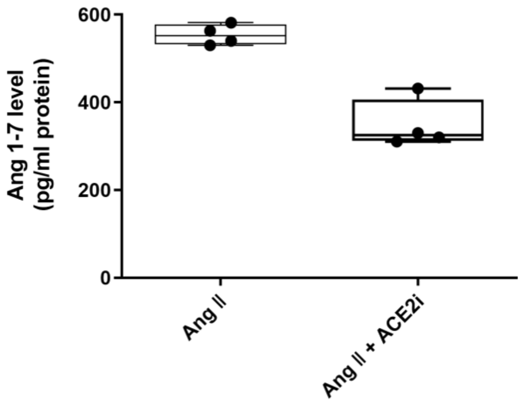

Supplementary Fig. 5. Ang 1-7 levels decreased after ACE2i treatment. Levels of Ang 1-7 were determined using ELISA in osteoclast culture media after administering Ang II (1×10^{-6} M) with or without ACE2i (1×10^{-5} M MLN-4760). ACE2i, angiotensin-converting enzyme 2 inhibitor; Ang, angiotensin.

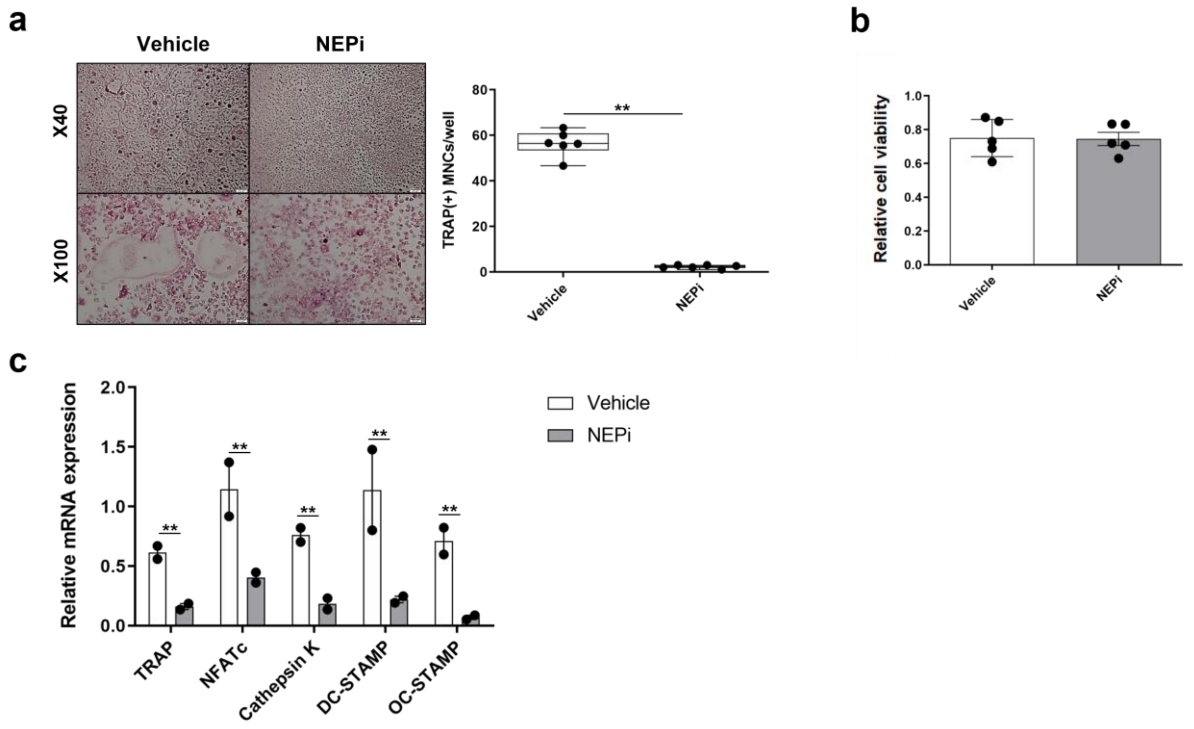

Supplementary Fig. 6. NEPi inhibited osteoclast differentiation. Mouse bone marrow macrophages were treated with or without NEPi (1×10^{-5} M sacubitrilat) (a) Representative TRAP staining images (left) and the number of TRAP-positive MNCs (>3 nuclei) per well (right). Scale bar = 50 µm. (b) CCK-8 assay. (c) RT-qPCR analysis. MNCs, multi-nuclear cells; NEPi, neprilysin inhibitor; TRAP, tartrate-resistant acid phosphatase. Data are presented as the mean ± SD.; $**p < 0.01$ using Mann-Whitney U test.

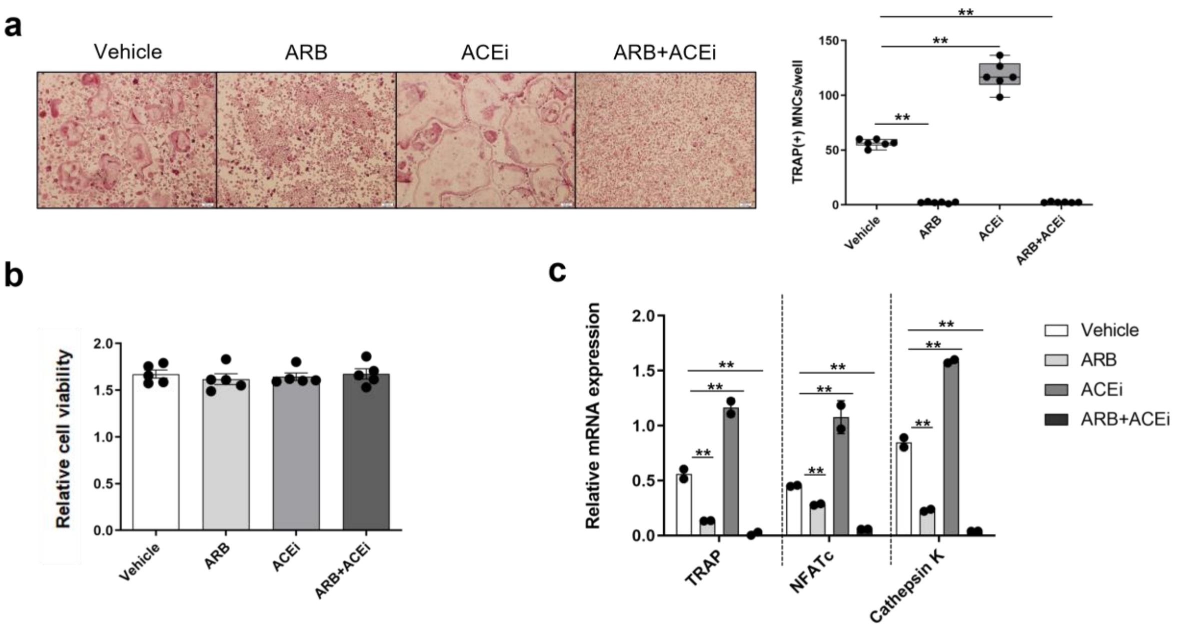

Supplementary Fig. 7. Treatment with combination of ARB and ACEi inhibited osteoclast differentiation. Mouse bone marrow macrophages were treated with ARB (1×10^{-5} M losartan) or ACEi (1×10^{-5} M captopril) or a combination of ARB and ACEi, and OCs were generated. (a) Representative TRAP staining images (left) and the number of TRAP-positive MNCs (>3 nuclei) per well (right). Scale bar = 50 µm. (b) CCK-8 assay. (c) RT-qPCR analysis. ACEi, angiotensin-converting enzyme inhibitor; ARB, angiotensin II receptor blocker; MNCs, multi-nuclear cells; TRAP, tartrate-resistant acid phosphatase. Data are presented as the mean ± SD.; $^{**}p < 0.01$ using Mann-Whitney U test.

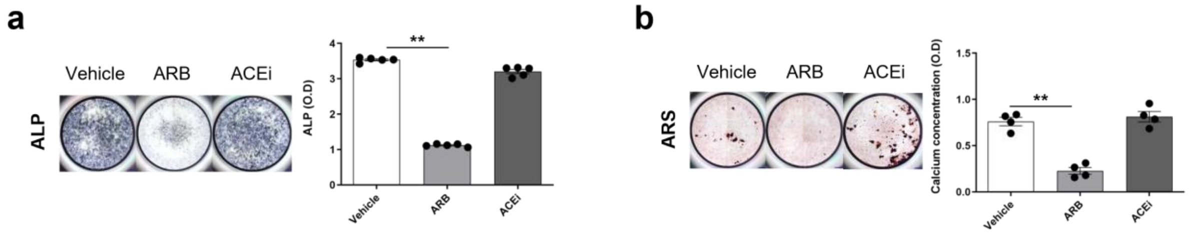

Supplementary Fig. 8. ARB, but not ACEi, inhibited osteoblast differentiation from SaOS2 cells. OBs were differentiated from SaOS2 cells in osteogenic medium with the vehicle, ARB (1×10^{-5} M losartan), or ACEi (1×10^{-5} M captopril). (a) ALP staining on day 7 (left) and ALP activity (right). (b) ARS on day 14 of osteoblast differentiation (left) and quantification of ARS (right). ACEi, angiotensin-converting enzyme inhibitor; ARB, angiotensin II receptor blocker; ALP, alkaline phosphatase; ARS, alizarin red. Data are presented as mean ± SD.; $**p < 0.01$ using Mann-Whitney U test.

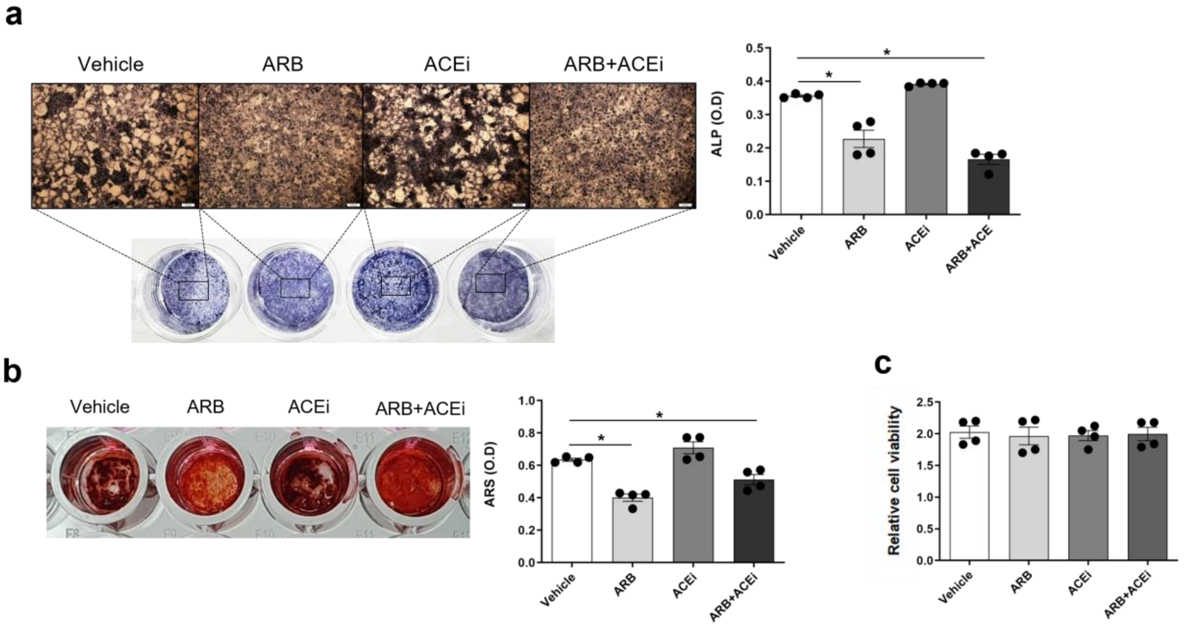

Supplementary Fig. 9. Treatment with combination of ARB and ACEi inhibited osteoblast differentiation. OBs were differentiated from mouse calvarial cells in osteogenic media with ARB (1×10^{-5} M losartan), ACEi (1×10^{-5} M captopril), or a combination of ARB and ACEi. (a) ALP staining on day 7 (left) and ALP activity (right). (b) ARS on day 21 of osteoblast differentiation (left) and its quantification (right). (c) CCK-8 assay. ACEi, angiotensin-converting enzyme inhibitor; ARB, angiotensin II receptor blocker; ALP, alkaline phosphatase; ARS, alizarin red; OB, osteoblast. Data are presented as the mean ± SD.; $*p <$ 0.05 using Mann-Whitney *U* test.

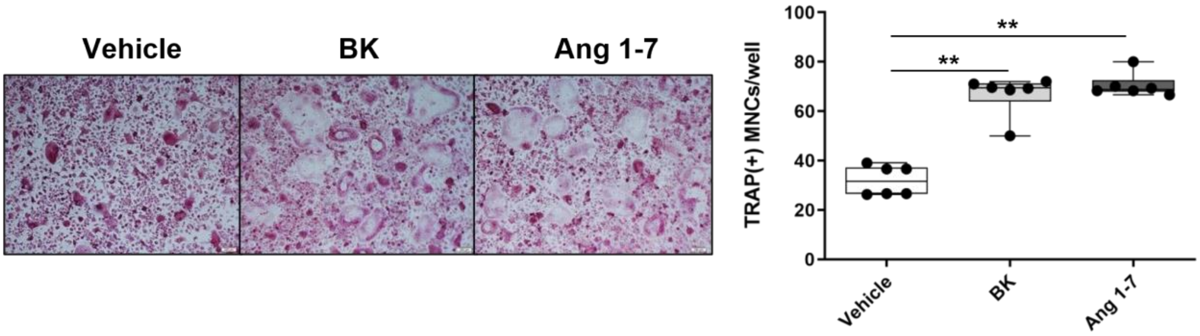

Supplementary Fig. 10. Bradykinin and Ang 1-7 promoted osteoclast differentiation. Mouse bone marrow macrophages were treated with BK (1×10^{-6} M) or Ang 1-7 (1×10^{-6} M), and OCs were generated. Representative TRAP staining images (left) and the number of TRAP-positive MNCs (>3 nuclei) per well (right). Scale bar = 50 µm. Ang 1-7, angiotensin 1-7; BK, bradykinin; MNCs, multi-nuclear cells; TRAP, tartrate-resistant acid phosphatase. Data are presented as mean ± SD.; $^{**}p < 0.01$ using Mann-Whitney *U* test.

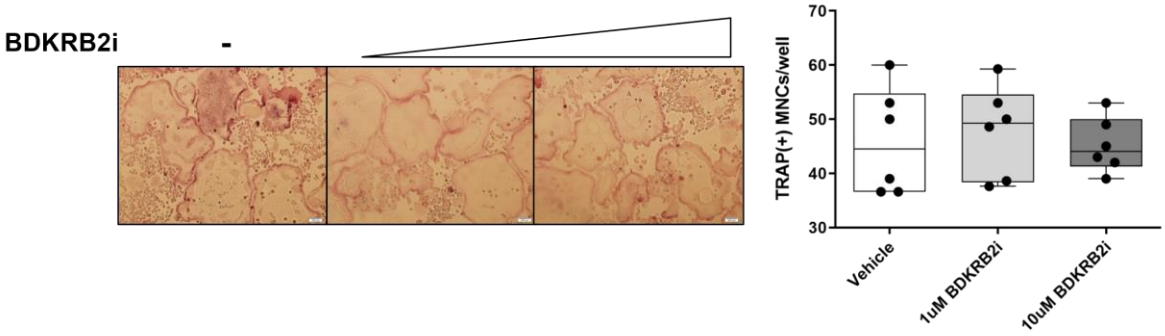

Supplementary Fig. 11. BDKRB2i did not affect osteoclast differentiation. Mouse bone marrow macrophages were treated with increasing doses of BDKRB2i, and OCs were generated. Representative TRAP staining images (left) and the number of TRAP-positive MNCs (>3 nuclei) per well (right). Scale bar = 50 µm. BDKRB2i, bradykinin receptor B2 inhibitor; MNCs, multi-nuclear cells; TRAP, tartrate-resistant acid phosphatase.

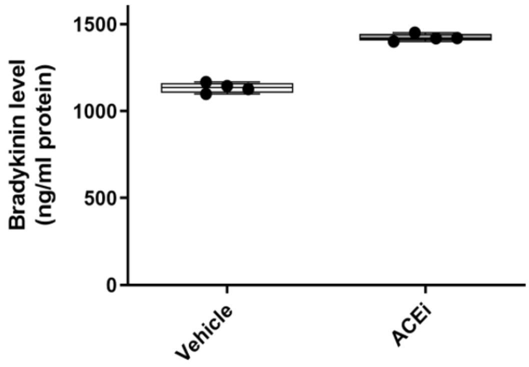

Supplementary Fig. 12. ACEi increased bradykinin levels. Bradykinin levels were measured using ELISA in OC culture media after administering ACEi (1×10^{-5} M captopril). ACEi, angiotensin-converting enzyme inhibitor.

Supplementary Table 1. Clinical characteristics of human blood samples

	**Control (n = 8)**	**r-axSpA (n = 6)**
Male (%)	8 (100)	6 (100)
Age ± SD	35.5 ± 3.4	33.8 ± 4.3
HLA-B27 positivity (%)	N/A	5 (83)
Disease duration, years ± SD	N/A	4.3 ± 2.5
Use of TNF inhibitor	N/A	N/A
ESR (mm/hr)	N/A	6.3 ± 5.2
CRP (mg/dL)	N/A	<0.8

ESR, erythrocyte sedimentation rate; CRP, C reactive protein; HLA-B27, human leucocyte antigen B27; N/A, not applicable; r-axSpA, radiographic axial spondyloarthritis

Supplementary Table 2. Clinical characteristics of human facet joint samples

	**Control**<br>**(n = 7)**	**r-axSpA**<br>**(n = 7)**
Male (%)	7 (100)	6 (85.7)
Age ± SD	51.3 ± 5.7	39.7 ± 5.7
HLA-B27 positivity (%)	N/A	7 (100)
Disease duration, years ± SD	N/A	20 ± 9.0
Use of TNF inhibitor	N/A	5 (71.4)
ESR (mm/hr)	N/A	25.9 ± 21.5
CRP (mg/dL)	N/A	<0.8

ESR, erythrocyte sedimentation rate; CRP, C reactive protein; HLA-B27, human leucocyte antigen B27; N/A, not applicable; r-axSpA, radiographic axial spondyloarthritis

Supplementary Table 3. Primers used in real-time PCR

Gene	Forward primer	Reverse primer
18s rRNA	5'-AACACGGGAAACCTCACCC-3'	5'-CCACCAACTAAGAACGGCCA-3'
mAGT	5'-CTGGATTTATCCACTGACCCAGTTC-3'	5'-TGGACTCCAGGCAGCTGAGA-3'
mACE	5'-CCACTATGGGTCCGAGTACATCAA-3'	5'-AGGGCGCCACCAAATCATAG-3'
mAT1R	5'-TGGGCGTCATCCATGACTGTA-3'	5'-TGAGTGCGACTTGGCCTTTG-3'
mAT2R	5'-GTGCATGCGGGAGCTGAGTA-3'	5'-ATTGGTGCCAGTTGCGTTGA-3'
mNEP	5'-AATGCTCCAAAGCCAAAGAA-3'	5'-CGATCATTGTCACCGCTATG-3'
HuPo	5'-CCATTCTATCATCAACGGGTACAA-3'	5'-AGCAAGTGGGAAGGTGTAATCC-3'
hAGT	5'-CGCCTGCCTGCTGCTGAT-3'	5'-GGAAAGTGAGACCCTCCACCTTGT-3'
hACE	5'-CAGGTGGTGTGGAACGAGTATGC-3'	5'-TCTCTGTGGTGATGTTGGTGTTGTAGT-3'
hAT1R	5'-GCCTCCTCGCCAATGATTCCA-3'	5'-CGTCCTGTCACTCGCTGCTG-3'
hAT2R	5'-CGCGAGAAGATGACCCAGAT-3'	5'-GAGTCCATCACGATGCCAGT-3'
hNEP	5`-GCCTCAGCCGAACCTACAAG-3`	5`-AATTTGCACAACGTCCTCAAGTT-3`
TRAP	5'-GCTGGAAACCATGATCACCT-3'	5'-GAGTTGCCACACAGCATCAC-3'
Cathepsin K	5'-AGGGAAGCAAGCACTGGATA-3'	5'-GCTGGCTGGAATCACATCTT-3'
NFATc1	5'-CTCGAAAGACAGCACTGGAGCAT-3'	5'-CGGCTGCCTTCCGTCTCATAG-3'
OC-STAMP	5'-ATGAGGACCATCAGGGCAGCCACG-3'	5'-GGAGAAGCTGGGTCAGTAGTTCGT-3'
DC-STAMP	5'-TCCTCCATGAACAAACAGTTCCAA-3'	5'-AGACGTGGTTTAGGAATGCAGCTC-3'
BMP2	5'-TCCGCTCCACAAACGAGAAA-3'	5'-AAAGGCATGATAGCCCGGAG-3'
Osterix	5'-AGCGACCACTTGAGCAAACAT-3'	5'-GCGGCTGATTGGCTTCT-3'
Osteocalcin	5'-CTGACCTCACAGATGCCAAG-3'	5'-GTAGCGCCGTGAGTCTGTTC-3'
RANKL	5'-TGTACTTTCGAGCGCAGATG-3'	5'-CCACAATGTGTTGCAGTTCC-3'
Runx2	5'-AGATGACATCCCCATCCATC-3'	5'-GTGAGGGATGAAATGCTTGG-3'
